# What goes around comes around: Artificial circular RNAs bypass cellular antiviral responses

**DOI:** 10.1016/j.omtn.2022.04.017

**Published:** 2022-04-27

**Authors:** Janina Breuer, Patrick Barth, Yannic Noe, Lyudmila Shalamova, Alexander Goesmann, Friedemann Weber, Oliver Rossbach

**Affiliations:** 1 Institute of Biochemistry, Faculty of Biology and Chemistry (FB08), Justus-Liebig-University of Giessen, Heinrich-Buff-Ring 17, 35392 Giessen, Germany; 2 Bioinformatics and Systems Biology, University of Giessen, Heinrich-Buff-Ring 58, 35392 Giessen, Germany; 3 Institute for Virology, Faculty of Veterinary Medicine, University of Giessen, Schubertstr. 81, 35392 Giessen, Germany

**Keywords:** MT: Non-coding RNAs, circular RNA, circular RNA sponges, innate immune response, immunogenicity, RNA therapy, molecular sponge, artificial circRNA, sensory molecules, RNA sensors

## Abstract

Natural circular RNAs have been found to sequester microRNAs and suppress their function. We have used this principle as a molecular tool and produced artificial circular RNA sponges in a cell-free system by *in vitro* transcription and ligation. Formerly, we were able to inhibit hepatitis C virus propagation by applying a circular RNA decoy strategy against microRNA-122, which is essential for the viral life cycle. In another proof-of-principle study, we used circular RNAs to sequester microRNA-21, an oncogenic and pro-proliferative microRNA. This strategy slowed tumor growth in a 3D cell culture model system, as well as in xenograft mice upon systemic delivery. In the wake of the global use of an *in vitro* transcribed RNA in coronavirus disease 2019 (COVID-19) vaccines, the question arose whether therapeutic circular RNAs trigger cellular antiviral defense mechanisms when delivered systemically. In this study, we present data on the cellular innate immune response as a consequence of liposome-based transfection of the circular RNA sponges we previously used to inhibit microRNA function. We find that circular RNAs produced by the presented methodology do not trigger the antiviral response and do not activate innate immune-signaling pathways.

## Graphical abstract

**Figure:**
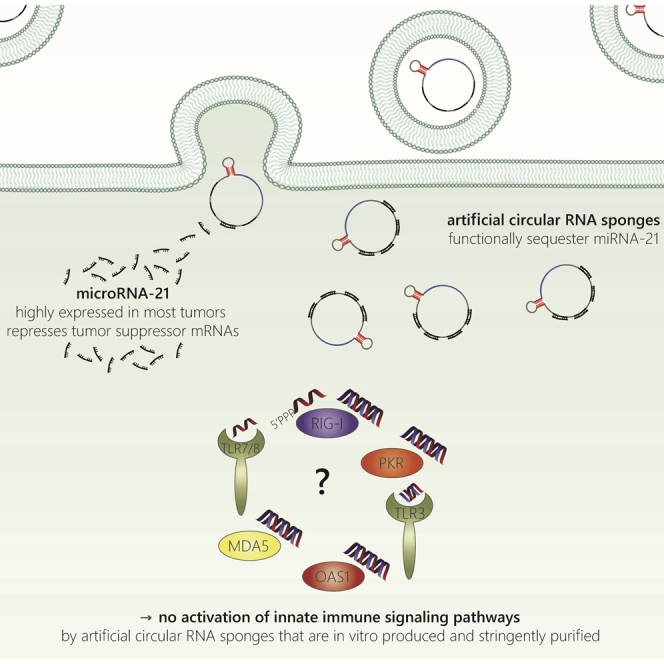

## Teaser

Circular RNAs produced by *in vitro* transcription can be used to inhibit microRNAs that are involved in viral or cancerous human diseases. Here, we show that circular RNAs are not sensed by the cell-intrinsic innate immune system, a crucial precondition for their clinical application.

## Introduction

Research on circular RNA (circRNA) has been underestimated and neglected for a long period but has become increasingly important for molecular biology, medicine, and pharmaceutical science in the recent years. Harboring neither a 5′ cap nor a 3′ poly(A) tail, circRNA molecules belong to a class of mainly non-coding RNAs.^1^, ^2^, ^3^, ^4^, ^5^ Naturally occurring circRNAs arise by an alternative splicing mechanism termed “back splicing”^3^ or “head-to-tail splicing.”^2^ This process is characterized by joining a splice donor to an upstream instead of a downstream splice acceptor site, relying on canonical splicing signals.^1^, ^2^, ^3^, ^4^, ^5^ This results in a covalently closed and therefore circularized structure, which is characterized by resistance to exonucleolytic degradation and an elevated stability compared with linear RNAs.^3^^,^^6^, ^7^, ^8^, ^9^

Even though naturally occurring circRNAs appear to be highly abundant, found in diverse species throughout the eukaryotic Tree of Life, and strictly regulated, the molecular function of most endogenous circRNAs is still unknown.^2^^,^^3^^,^^6^^,^^10^, ^11^, ^12^ Nevertheless, there are several lines of evidence showing that circRNAs have important regulatory roles in cell biology and development.^2^^,^^11^^,^^13^, ^14^, ^15^ As described by Holdt et al.^16^ in 2017, circRNAs function in two general ways. First underlies the process of circRNA generation itself, and second, following their formation, their function as *trans*-acting molecule. Considering the latter, recent studies demonstrate that naturally occurring circRNAs have at least three major functions in eukaryotic cells: circRNAs can function as microRNA (miRNA) sponges, interact with RNA-binding proteins (RBPs), and act as nuclear transcriptional regulators—all together illustrating the impact of circRNAs on the regulatory networks governing gene expression.^15^^,^^17^^,^^18^

When binding cytoplasmic miRNAs as molecular sponges, circRNAs are able to regulate and neutralize endogenous miRNA levels. In this context, two initial studies, published simultaneously in 2013 by the groups of Nikolaus Rajewsky and Jørgen Kjems, focused on the *cerebellar degeneration*-*related protein 1 transcript* (*CDR1as*) gene, which expresses the cellular circRNA CDR1as/ciRS-7. CDR1as/ciRS-7 acts as a molecular sponge sequestering the cytoplasmic miRNA-7 via 70 highly conserved binding sites, hence its alternative name circRNA sponge for miRNA-7 (miR-7) (ciRS-7).^2^^,^^12^ Co-expressed with miRNA-7 in neocortical and hippocampal neurons, CDR1as/ciRS-7 leads to a sequestration of the miRNA, a suppression of miR-7 functions, and thereby a de-repression of natural miR-7 targets.^2^^,^^12^ CDR1as/ciRS-7 knockout mice showed deregulated miRNAs, dysfunctional synaptic transmission, and symptoms associated with human neuropsychiatric disorders.^19^ These exemplary results not only belay the sequestration of miRNAs by circRNAs and therefore the biological availability and function but also indicate the importance of circRNAs for normal cell functions.^16^

The described characteristics, functions, and importance of naturally occurring circRNAs imply the increasing relevance of artificially produced circRNAs as potential tool for molecular biology and medicine.^2^^,^^8^^,^^11^^,^^13^, ^14^, ^15^^,^^20^ In 2020, our group developed and published an optimized method for the engineering, *in vitro* production, and stringent purification of artificial ciRSs as novel agents for miRNA inhibition.^20^ This technique was firstly utilized in the hepatitis C virus (HCV) model system to provide a proof-of-principle study that artificial ciRSs are capable of the sequestration of mature miRNAs to impair their biological functions.^8^ Binding of miR-122 at two distinct binding sites at the 5′ end of the positive-sense, single-stranded RNA genome of the hepatocyte-specific virus results in the protection of the viral RNA from exonucleolytic degradation and enhancement of viral translation.^21^, ^22^, ^23^, ^24^ Miravirsen, a locked nucleic acid (LNA)-containing DNA oligonucleotide antisense to miR-122, inhibits viral propagation by sequestration of the miRNA.^25^ In this context, engineered ciRSs efficiently bound and sequestered the cellular miR-122 *in vitro* and *in vivo*, thereby reducing viral intracellular protein levels similar to Miravirsen.^8^

Moreover, the concept of artificial circRNA sponges was used to target miR-21. miR-21 is one of the earliest identified oncogenic miRNAs.^26^ When upregulated in cancer cells, miR-21 inhibits numerous tumor suppressor mRNAs, which is associated with proliferation, apoptosis, and invasion.^26^, ^27^, ^28^ As miR-21 is the most abundant miRNA across cancer transcriptomes, and *MIR21* knockout resulted in a reduced tumor growth, ciRSs were designed to impair miR-21 activities *in vivo*.^9^ In addition to causing a significant reduction in cell proliferation and invasion of 3D spheroid model systems, artificial ciRS sequestrating miR-21 significantly inhibited tumor growth in a lung adenocarcinoma xenograft mouse model via the upregulation of tumor-suppressor expression.^9^ Furthermore, the transfection of related circRNAs sequestering miR-21 led to induced apoptosis and increased expression of miR-21-regulated proteins in gastric cancer cell lines in another study.^29^

Two fundamental parameters determine the fate of any pharmaceutical: (1) the efficacy and (2) the toxicity.^30^ Considering the described findings, the past few years have witnessed the advent of artificial circRNA sponges as a novel and powerful antisense approach to inhibit miRNA activities in the context of human diseases, e.g., caused by viral infections or cancer.^8^^,^^9^^,^^29^^,^^30^ What remained controversial, however, was the question whether circRNAs may provoke innate immunity responses. The cellular innate immune system is able to detect foreign RNA via sensory molecules like RIG-I, PKR, or TLR7/8. This leads to the activation of various signaling pathways, resulting in the induction of cytokines, chemokines, and interferons.^31^ As recently also seen in the context of mRNA vaccines, the bypassing of the cellular immunity is crucial for the success of a clinical application.^32^ Several groups have tested circRNAs that have been artificially produced and purified by different procedures for their capability to stimulate RNA recognition pathways, with varying and controversial results.^33^^,^^34^

In this study, we demonstrate that artificial circRNAs can be utilized as miRNA sponges, if produced in a cell-free system by *in vitro* transcription and ligation, and stringently purified by gel extraction, can bypass the cellular RNA sensors, and are not recognized by the innate immune system.

## Results

### Artificial circular RNA sponges can be produced and purified *in vitro*

Encouraged by naturally occurring circRNAs containing binding sites that are able to sequester specific miRNAs, artificial ciRSs were designed to target the oncogenic miR-21.^2^^,^^9^^,^^12^ Together with a double-stranded 11-nt stem loop and a 63-nt constant region, the engineering process resulted in ciRS containing either four bulged miR-21-binding sites (ciRS-21-bulge [ciRS-21-bu]) or a randomized sequence with the exact same nucleotide composition (ciRS-21-random [ciRS-21-rnd]) as negative control for miRNA binding. Our earlier studies demonstrated that sequestration of miR-21 was highly efficient, resulting in an impaired oncogenic potential of cancer cells in cell culture and in a xenograft mouse model, as published in Müller et al.^9^ in 2020. Inspired by rising questions on the immunogenicity of circRNAs, this study focuses on the innate immune responses triggered by the same artificial ciRS.

**Figure 1:**
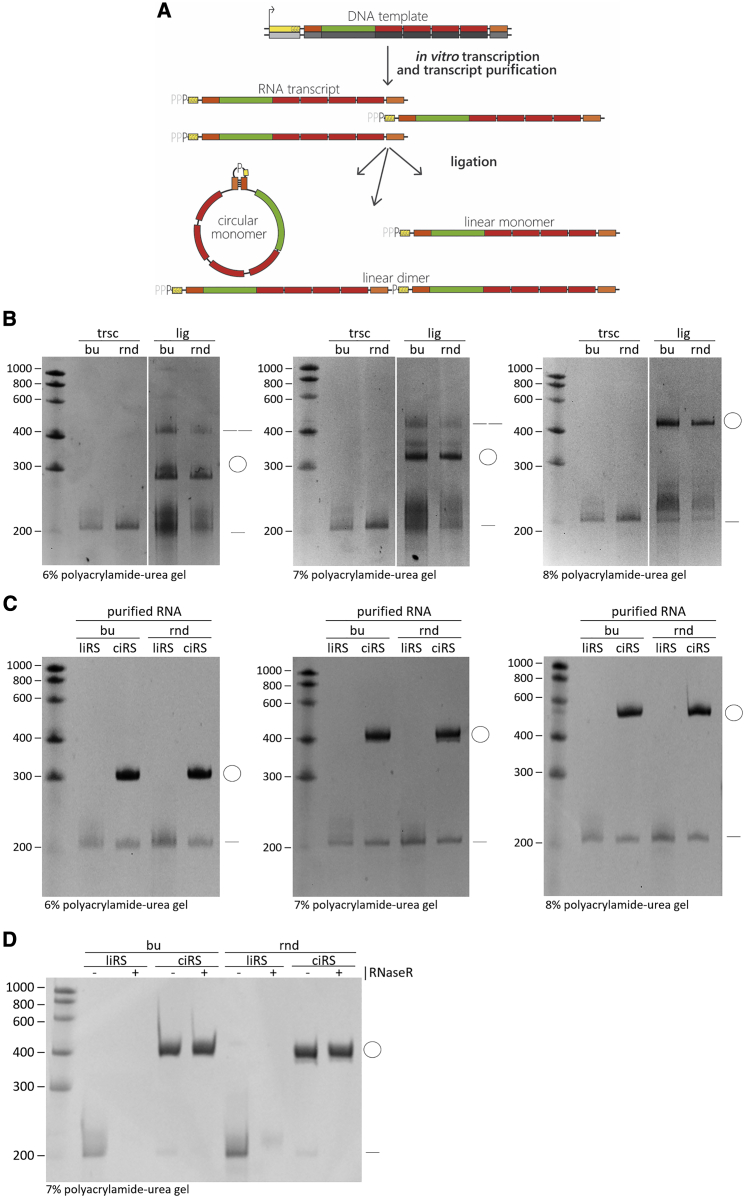
*In vitro* production of artificial circular RNA sponges (A) Schematic workflow of the ciRS production procedure—including an *in vitro* transcription from a DNA template, followed by transcript purification and ligation. Considering the activity of the T4 RNA ligase, transcript ligation resulted in both linear monomers and dimers of the transcript and circularized transcripts (indicated by the dash and double dash or the circle in B, C, and D). (B) *In vitro* transcription (trsc) and circularization (lig) reaction of constructs ciRS-21-bulge (bu) and ciRS-21-random (rnd) were analyzed by 6%, 7%, and 8% polyacrylamide-urea gel electrophoresis, followed by ethidium bromide staining. Linear and circular isoforms of the 208-nt transcripts show a circularization efficiency of ∼40%–80%, and only small amounts of linear dimers are detectable. While the mobility of linear RNAs remains unchanged compared with the RNA ladder, the mobility of circular RNA appears lower in higher percentage polyacrylamide-urea gels, resulting in a shift of the circular RNA when comparing the polyacrylamide-urea gels with different concentrations and allowing circRNA-specific gel purification. (C) Circular and linear isoforms of the RNA sponges (ciRS and liRS) were purified, and quality was verified on analytic 6% and 7% polyacrylamide-urea gels by ethidium bromide staining. (D) Both purified ciRS and liRS were subjected to RNase R exonuclease (+) or control (−) treatment and analyzed on 7% polyacrylamide-urea gel by ethidium bromide staining to validate circularity.

Our *in vitro* circRNAs production procedure, as described in detail elsewhere,^20^ includes three main steps: (1) the T7 RNA polymerase-mediated *in vitr*o transcription and transcript purification, (2) the transcript ligation based on the T4 RNA ligase, and (3) the gel purification of circular and linear constructs (Figure 1A). RNA quality and purity was controlled after each step of the production procedure (Figures 1B and 1C). After *in vitro* ligation, transcripts either remain linear monomers; are ligated intermolecularly, forming linear dimers or multimers; or are ligated intramolecularly into circular monomers (Figures 1A and 1B). As already reported in 1988 by Tabak et al.,^35^ the aberrant mobility of circRNA molecules within 6%, 7%, and 8% polyacrylamide-urea gels allows specific identification and separation and therefore enables gel purification of circular and linear isoforms of the RNA sponges (ciRSs and liRSs). While the relative mobility of linear RNAs remains unchanged compared with the respective RNA ladder, the mobility of circRNAs appears lower in higher percentage polyacrylamide-urea gels, resulting in a shift of the circRNA compared with marker bands (Figures 1B and 1C). ciRS-21-bu and ciRS-21-rnd show circularization efficiencies of 70%–80%. Other isoforms, as linear transcript monomers or dimers, were detectable but at substantially lower abundance (Figure 1B). RNase R exonuclease treatment of gel-purified circular and linear isoforms of ciRS-21-bu and ciRS-21-rnd verified circularity (Figure 1D).

### Differential expression analysis after ciRS transfection

**Figure 2:**
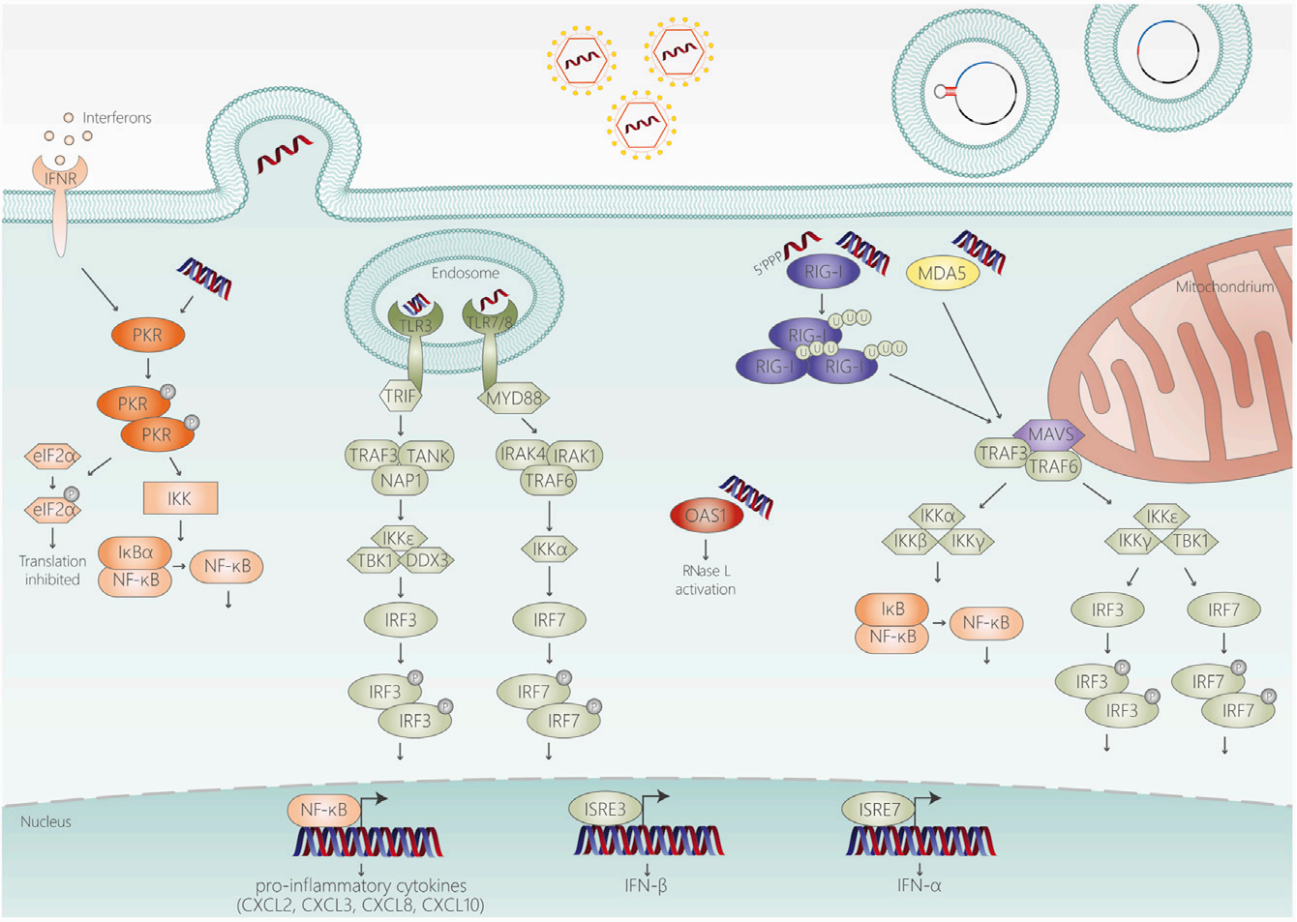
Cellular mechanisms for identifying foreign RNAs The innate immunity is the first line of defense against invading pathogens entering a cell. In this context, cell-associated pattern recognition receptors are crucial for the detection of pathogen-associated molecular patterns (PAMPs), including, for example, viral single- or double-stranded RNA within the endosome or cytoplasm of a cell. Consequently, these intracellular sensory molecules like PKR, TLR3, TLR7, TLR8, OAS1, RIG-I, and MDA5 lead to an activation of a cascade of downstream signaling pathways, triggering an inflammatory response by inducing cytokines, chemokines, and interferons. However, the mechanism detecting foreign circular RNA is still unclear.

**Figure 3:**
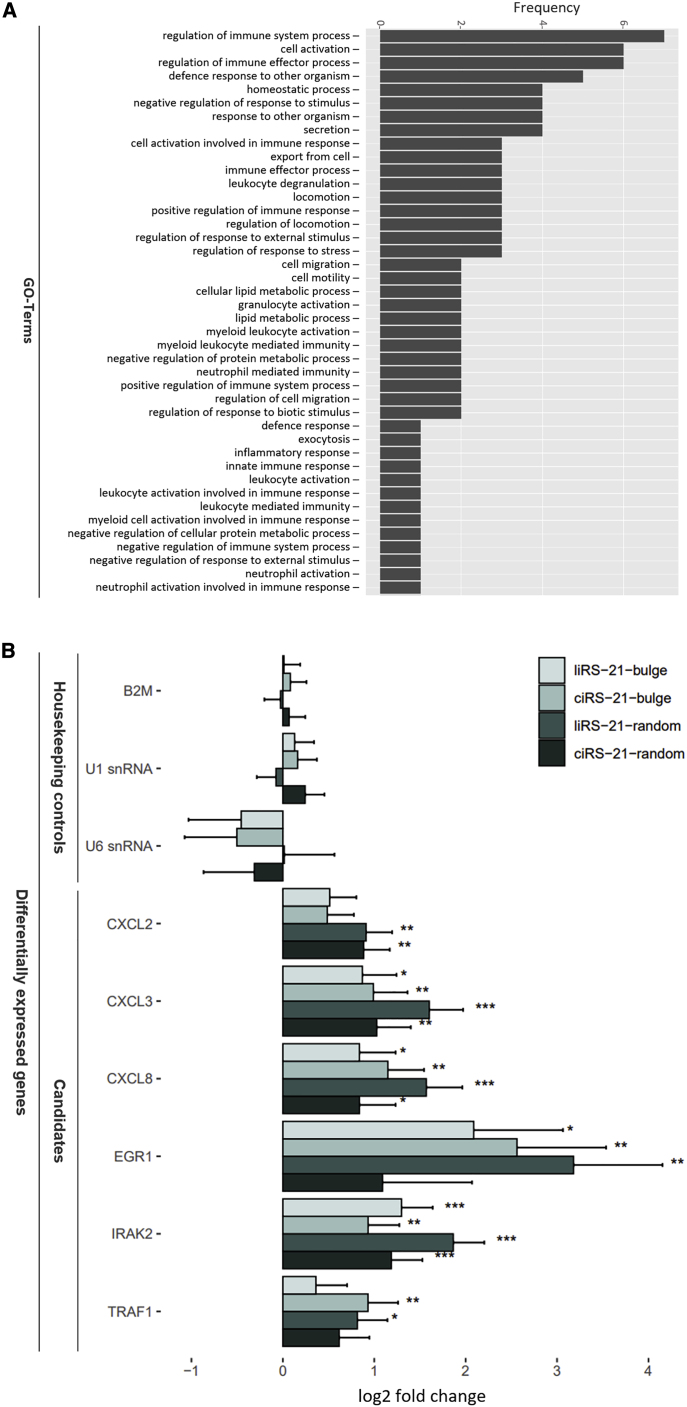
RNA sequencing revealed low immunogenic potential of ciRS-21-bu and ciRS-21-rnd and their linear counterparts A549 cells were transfected with 250 ng of the artificial linear and circular RNA sponges ciRS-21-bu and ciRS-21-rnd. At 3 h post-transfection, A549 cells were harvested, total RNA was isolated, and RNA sequencing was performed. Upregulated mRNAs upon ciRS-21-bu transfection were subjected to (A) Gene Ontology (GO) term analysis. (B) Furthermore, *CXCL2*, *CXCL3*, *CXCL8*, *EGR1*, *IRAK2*, and *TRAF1* were identified as differentially expressed candidate mRNAs upon ciRS-21-bu and ciRS-21-rnd transfection relative to mock-treated cells incubated with the transfection reagent only. *B2M* mRNA, U1 snRNA, and U6 snRNA were used as housekeeping controls. Statistical significance indicated by p values (∗p < 0.05; ∗∗p < 0.01; ∗∗∗p < 0.001).

Foreign RNA, e.g., viral RNA, is efficiently detected by the host antiviral innate immune system.^36^ The array of RNA sensors includes single- and double-stranded RNA-binding proteins, such as RIG-I, PKR, and the TLRs (Figure 2).^37^^,^^38^ However, despite several publications discussing roles of RIG-I and PKR in detection of foreign circRNA molecules, the cellular mechanisms of discrimination and detection of foreign circRNAs are largely unknown.^33^^,^^34^^,^^39^^,^^40^ Aiming to identify pathways activated after transfection of the linear and circular miR-21 (liRS/ciRS-21-bu) and control RNAs (liRS/ciRS-21-rnd) into A549 cells, transcriptome analysis was performed via RNA sequencing (Figure 3). Gene ontology (GO) enrichment analysis of genes more than 4-fold upregulated after ciRS-21-bu treatment indicated a variety of processes, such as the regulation of immune system process, cell activation, and secretion (Figure 3A). Differential expression analysis revealed that the transcriptome of cells transfected with ciRS/liRS-21-bu or ciRS/liRS-21-rnd was very similar to cells without RNA transfection, with only a few differentially expressed mRNAs detectable. Nevertheless, six potential candidates upregulated after ciRS transfection could be determined in context of the innate immune response: *CXCL2*, *CXCL3*, *CXCL8*, *EGR1*, *IRAK2*, and *TRAF1* (Figure 3B), although the increase in expression was moderate (2- to 4-fold) in the differential expression analysis.

### Innate immune responses can be stimulated with an extensively double-stranded circRNA

**Figure 4:**
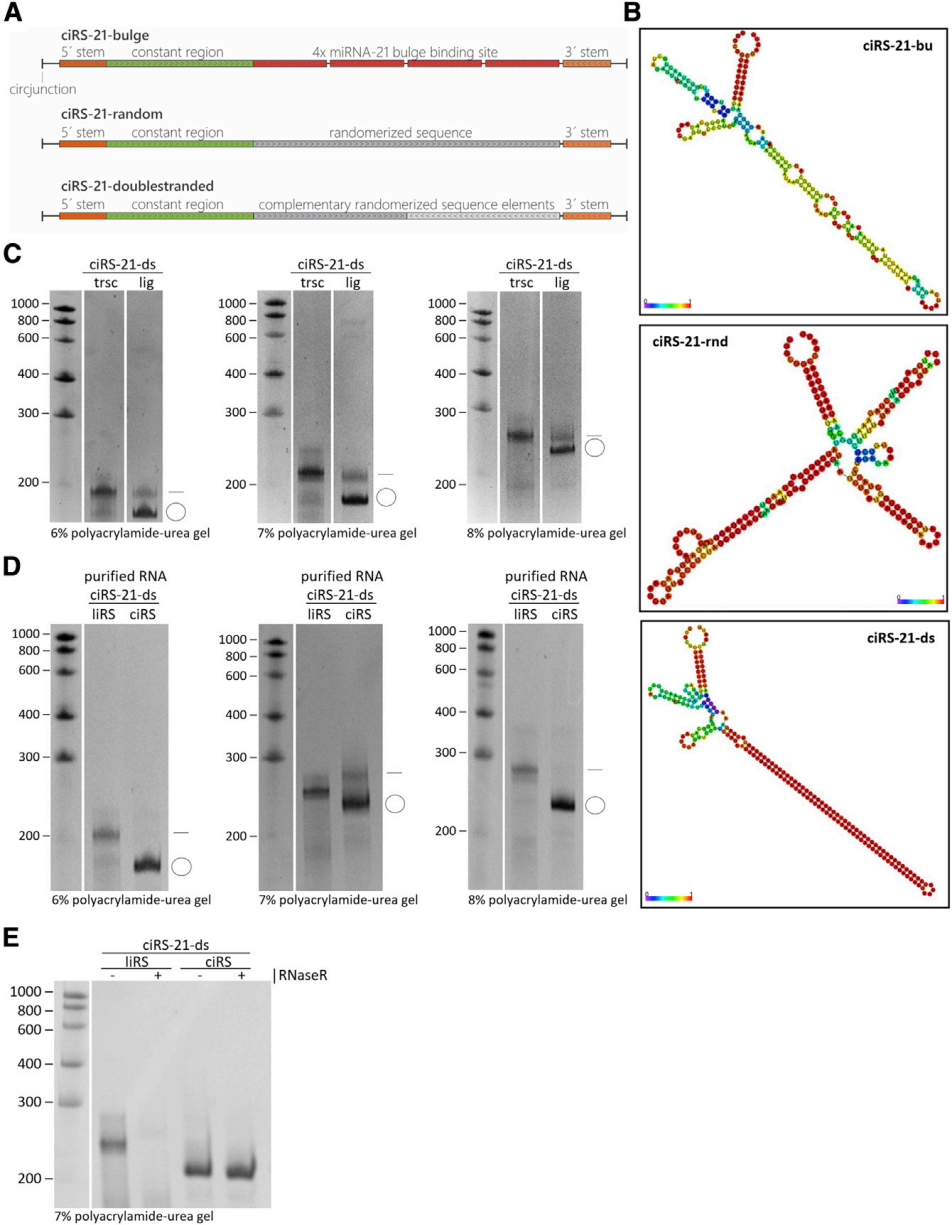
Design and *in vitro* production of a highly double-stranded artificial circular RNA sponge (A) Schematic overview of structure and sequence properties of the analyzed artificial circular RNA sponges with a length of 208 nt. Conventionally, all transcripts share several sequence elements: a 5′ and 3′ stem (orange) sequence forming an 11-nt double-stranded stem-loop structure and a 63-nt “constant region” (green), which serves as binding site for, e.g., PCR primers or antisense probes in northern blotting. The ciRS-21-bu includes four miR-21-binding sites (red) containing a bulge between nucleotides 10 and 12 and a spacing of 4 nt between binding sites. Contrastingly, ciRS-21-rnd is characterized by a randomized sequence (dark gray), and based on the latter, ciRS-21-ds contains a double-stranded randomized sequence (dark and light gray) as central element. Vertical bars (|) mark the circular junctions. Less than (<) and greater than (>) symbols indicate complementary sequence elements. (B) Secondary structure of ciRS-21-bu, ciRS-21-rnd, and ciRS-21-ds predicted by RNAfold 2.4.18 online tool by calculation of the minimum free energy of the RNA sequence. Predicted strong secondary structure interactions are indicated in red; low probability interactions are indicated in blue. (C) Analysis of the *in vitro* transcription (trsc) and ligation (lig) of the highly double-stranded ciRS-21-ds via 6%, 7%, and 8% polyacrylamide-urea gel electrophoresis, followed by ethidium bromide staining. Linear and circular transcripts (indicated by the dash or the circle, respectively) display an aberrant mobility of the 208 nt comprising construct, and a circularization efficiency is estimated at ∼80%. (D) Circular and linear isoforms of the RNA sponge (ciRS-21-ds and liRS-21-ds) were gel purified, and RNA integrity was verified on analytic 6% and 7% polyacrylamide-urea gels by ethidium bromide staining. (E) Both purified ciRS and liRS were subjected to RNase R exonuclease (+) or control (−) treatment and analyzed on 7% polyacrylamide-urea gel by ethidium bromide staining to validate circularity.

As the sequencing results indicated a surprisingly low extent of innate immunity activation, we included a set of control RNA molecules for immune activation. Apart from using only known immunostimulants, such as high- and low-molecular-weight polyinosinic-polycytidylic acid (HMW and LMW poly(I:C)), which mimic long double-stranded RNAs (dsRNAs), and single-stranded poly uridine (ssPolyU), we created an extensively double-stranded circRNA. In more detail, we engineered an artificial ciRS sharing the same basic structure as ciRS-21-bu and ciRS-21-rnd (stem loop and constant region) but containing a randomized self-complementary sequence of 50 bp, resulting in ciRS-21-double-stranded (ciRS-21-ds) (Figure 4A). The reverse-complementary sequence element of ciRS-21-ds was designed based on the nucleotide composition of the randomized ciRS-21-rnd circRNA (Figure 4A). Compared with ciRS-21-bu and ciRS-21-rnd, RNAfold secondary structure prediction for ciRS-21-ds showed the high-confidence base-pairing interaction within the sequence, with a maximum of 50 bp, resulting in a secondary structure formation (Figure 4B). As reported for ciRS-21-bu and ciRS-21-rnd, ciRS-21-ds was produced via *in vitro* transcription, ligation of the transcripts, and gel purification of linear and circular isoforms (Figures 1A, 4C, and 4D). Contrary to previously illustrated *in vitro* ligations (Figure 1),^8^^,^^9^^,^^20^ production of ciRS-21-ds was less efficient. The highly double-stranded sequence of this construct affected the transcription efficiency and altered the mobility within polyacrylamide-urea gels. As seen in Figures 4C and 4D, not only circular but also linear constructs of ciRS-21-ds showed a size shift compared with the RNA ladder within 6%, 7%, and 8% polyacrylamide-urea gels, making it difficult to distinguish linear and circular isoforms. Surprisingly, highly double-stranded ciRS-21-ds migrated even faster than liRS-21-ds in 6%, 7%, and 8% polyacrylamide-urea gels (Figure 4C). Notably, no linear dimers of intermolecularly ligated transcripts were detected and the observed circularization efficiency was 90% compared with remaining linear transcript monomers (Figure 4C). Analytic polyacrylamide-urea gels and RNase R exonuclease treatment of purified liRS and ciRS confirmed that, despite the difficulties in identification of circular and linear isoforms within polyacrylamide-urea gels, the purification of ciRS-21-ds was successful (Figures 4D and 4E).

### ciRS-21-bu and ciRS-21-rnd do not trigger the cellular innate immune response

**Figure 5:**
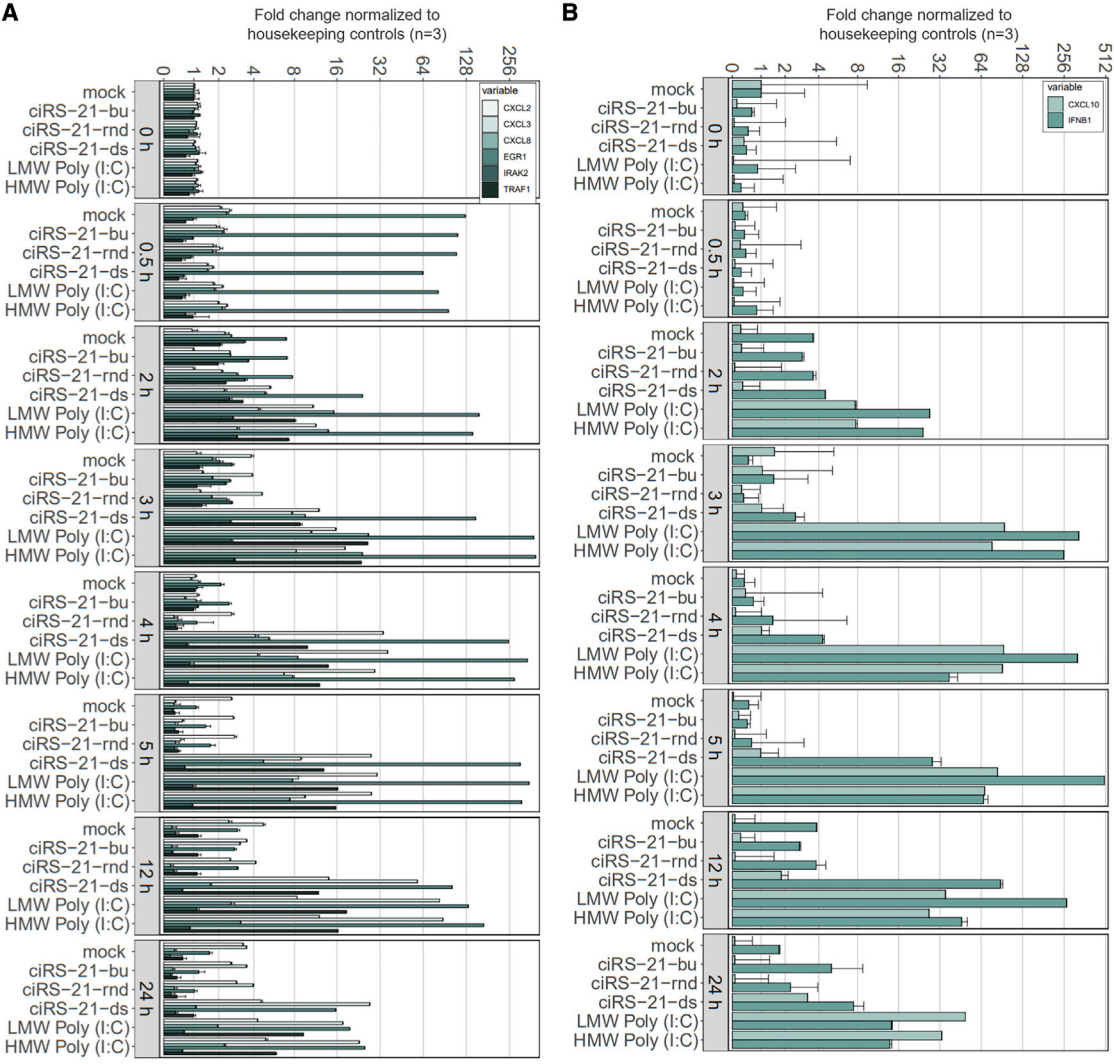
Time-dependent immune response to ciRS-21-ds and poly(I:C) in contrast to ciRS-21-rnd and ciRS-21-bu treatment A549 cells were transfected with 250 ng of either the artificial circular RNA sponges, ciRS-21-bu, ciRS-21-rnd, and ciRS-21-ds or with the immunostimulants low-molecular-weight and high-molecular-weight polyinosinic:polycytidylic acid (LMW poly(I:C) and HMW poly(I:C)). As control, cells were only treated with the transfection reagent (mock) but no circular RNA or immunostimulant. We harvested A549 cells 0, 0.5, 2, 3, 4, 5, 12, and 24 h post transfection, and total RNA was isolated. qRT-PCR was performed for the candidates upregulated in RNA sequencing: (A) *CXCL2*, *CXCL3*, *CXCL8*, *IRAK2*, *EGR1*, and *TRAF1* as well as for (B) *CXCL10* and *IFNB1*. Data were normalized to mean value of *B2M* mRNA, U1 snRNA, and U6 snRNA housekeeping controls. Error bars represent SD (n = 3). Data shown are representative of three independent experiments. Statistical significance, indicated by p values, was determined by Student’s t test (Tables S2 and S3). The numeric fold change of the mRNA expression of candidate genes is displayed using a logarithmic scale.

**Figure 6:**
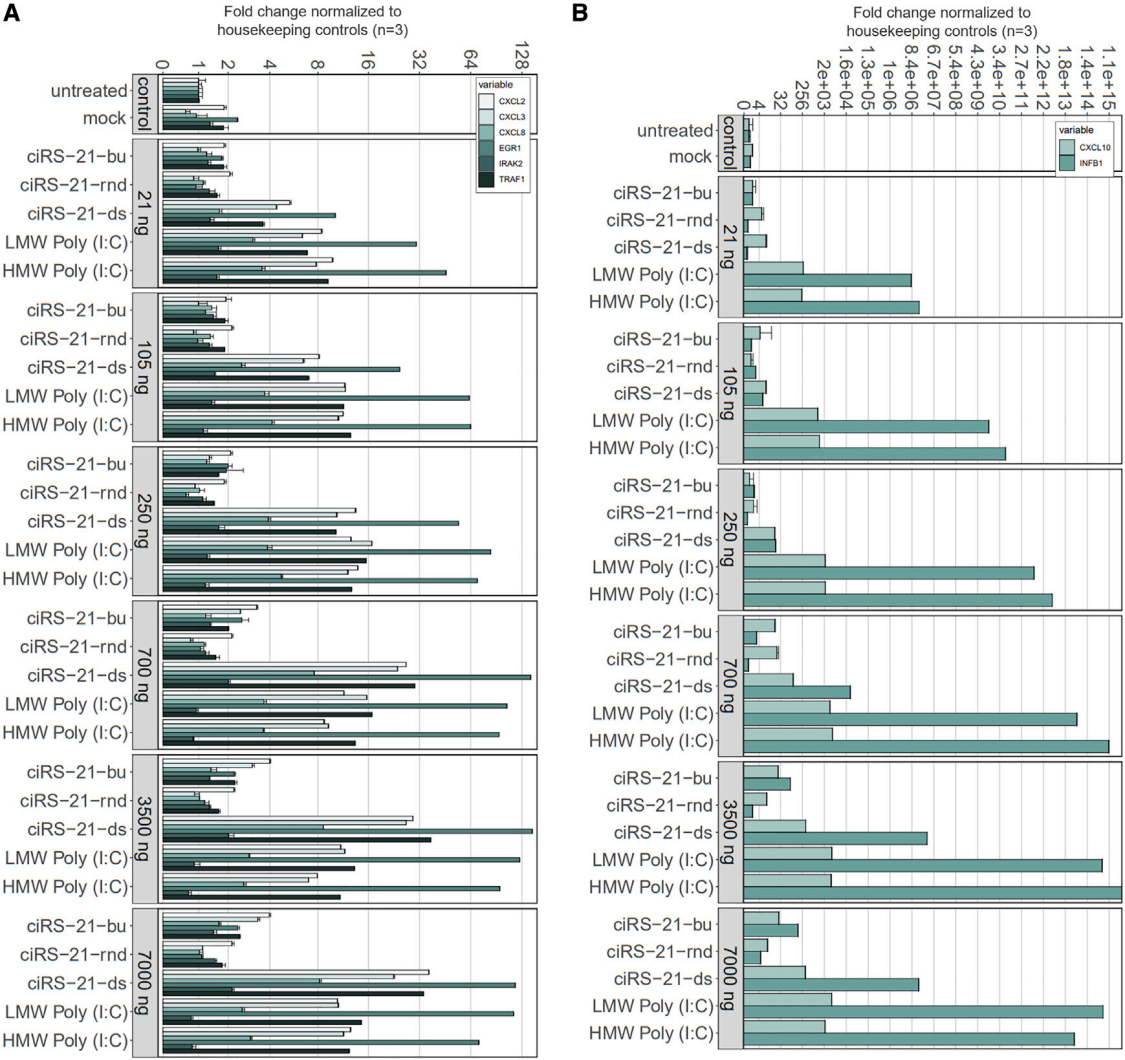
Dose-dependent immune activation to ciRS-21-ds and poly(I:C) in contrast to ciRS-21-rnd and ciRS-21-bu treatment A549 cells were transfected with 21, 105, 250, 700, 3,500, or 7,000 ng of either the artificial circular RNA sponges, ciRS-21-bu, ciRS-21-rnd, and ciRS-21-ds or with the immunostimulants LMW poly(I:C) and HMW poly(I:C). As control, cells were only treated with the transfection reagent (mock) but no circular RNA or immunostimulant. Three hours post-transfection, A549 cells were harvested and total RNA was isolated. qRT-PCR was performed for the candidate mRNAs derived from RNA sequencing: (A) *CXCL2*, *CXCL3*, *CXCL8*, *IRAK2*, *EGR1*, and *TRAF1* as well as for (B) *CXCL10* and *IFNB1*. Data were normalized to mean value of *B2M* mRNA, U1 snRNA, and U6 snRNA housekeeping controls. Error bars represent SD (n = 3). Data shown are representative of three independent experiments. Statistical significance, indicated by p values, was determined by Student’s t test (Tables S4 and S5). The numeric fold change of the mRNA expression of candidate genes is displayed using a logarithmic scale.

**Figure 7:**
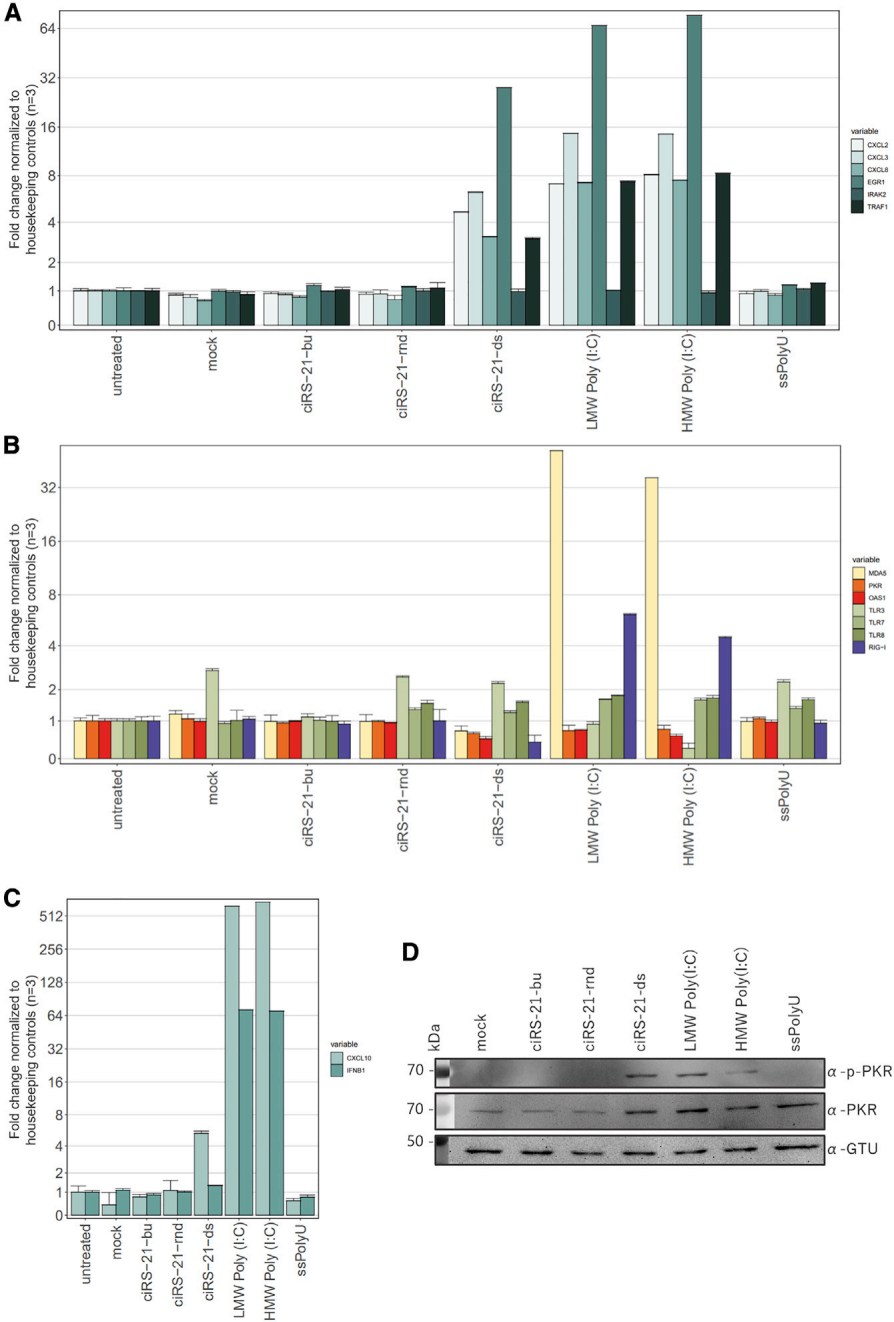
PKR is activated after ciRS-21-ds or poly(I:C) treatment A549 cells were transfected with 250 ng of either the artificial circular RNA sponges, ciRS-21-bu, ciRS-21-rnd, and ciRS-21-ds or with the immunostimulants LMW poly(I:C) and HMW poly(I:C). As control, cells were only treated with the transfection reagent (mock) but no circular RNA or immunostimulant. Three hours post-transfection, A549 cells were harvested and total RNA was isolated. qRT-PCR was performed for the candidates upregulated in RNA sequencing: (A) *CXCL2*, *CXCL3*, *CXCL8*, *IRAK2*, *EGR1*, and *TRAF1* as well as for mRNAs of cellular RNA sensors (B) *MDA5*, *PKR*, *OAS1*, *TLR3*, *TLR7*, *TLR8*, and *RIG-I* and the known downstream components of immune response (C) *CXCL10* and *IFNB1*. Data were normalized to mean value of *B2M* mRNA, U1 snRNA, and U6 snRNA housekeeping controls. Error bars represent SD (n = 3). Statistical significance, indicated by p values, was determined by Student’s t test (Table S6). (D) A549 cells were harvested 3 h post-transfection with 250 ng of ciRS-21-bu, ciRS-21-rnd, ciRS-21-ds, LMW poly(I:C), HMW poly(I:C), or single-stranded PolyU (ssPolyU), and total protein was isolated. Activated Thr466-phophorylated PKR (p-PKR) and total PKR as well as γ-tubulin as housekeeping control were analyzed by immunoblotting. Data shown are representative of three independent experiments. The numeric fold change of the mRNA expression of candidate genes is displayed using a logarithmic scale.

Next, effects of ciRS-21-bu, ciRS-21-rnd, and ciRS-21-ds transfections of A549 cells were analyzed compared with cells treated with immunostimulants, such as HMW or LMW poly(I:C) and ssPolyU, or liposome transfected control (mock) cells (Figures 5, 6, and 7). We analyzed mRNA levels of the candidate regulated mRNAs of *CXCL2*, *CXCL3*, *CXCL8*, *EGR1*, *IRAK2*, and *TRAF1* identified in RNA sequencing (Figure 3) via qRT-PCR (Figures 5A, 6A, and 7A). Immunogenicity of transfected constructs was additionally assessed via the chemokine *CXCL10* and the interferon *INFB1* mRNA levels (Figures 5B, 6B, and 7C). After normalization to mock-treated control cells, only a global, unspecific response was observed in all transfections after 0.5 h, regardless of the agents used. However, a more specific response to ciRS-21-ds and both poly(I:C) controls manifested within 2–24 h (Figure 5). In addition, cellular response to treatment with increasing doses of the different constructs varying from 21 ng to 7,000 ng was analyzed 3 h after transfection of A549 cells. Even minimal amounts of ciRS-21-ds, HMW poly(I:C), and LMW poly(I:C) led to a potent increase in the levels of mRNAs associated with innate immunity signaling, as detected by qRT-PCR (Figure 6). However, treatment of A549 cells with ciRS-21-bu resulted in minimal upregulation of these mRNAs and only after treatment with high doses of the circRNA (Figure 6).

In all experiments performed, cellular response to the highly double-stranded ciRS-21-ds was found to mimic immunogenicity of poly(I:C) treatment (Figures 5, 6, and 7). Therefore, within this study, we reveal a time- and dose-dependent immunogenicity of ciRS-21-ds, but not of ciRS-21-bu or ciRS-21-rnd—as evident from an upregulation of *CXCL2*, *CXCL3*, *CXCL8*, *EGR1*, and *TRAF1* mRNAs, as well as *CXCL10* and *INFB1* (Figures 5, 6, and 7). However, *IRAK2* mRNA levels remained unchanged.

To identify a potential upregulation of sensory molecules involved in circRNA sensing (Figure 2), we performed qRT-PCR to analyze the mRNA levels of *MDA5*, *PKR*, *OAS1*, *TLR3*, *TLR7*, *TLR8*, and *RIG-I* after transfection (Figure 7B). Despite an increase of *MDA5* and *RIG-I* mRNA levels upon HMW poly(I:C) and LMW poly(I:C) treatment, no transcriptional upregulation of RNA sensors could be detected after circRNA transfection (Figure 7B). Nonetheless, immunoblotting analysis of PKR in A549 cells revealed PKR activation by detection of phosphorylated PKR (Thr446 p-PKR) 3 h after transfection with 250 ng of ciRS-21-ds, HMW poly(I:C), and LMW poly(I:C), but not of ciRS-21-bu, ciRS-21-rnd, or ssPolyU. We conclude that our artificial circRNAs produced by *in vitro* transcription and purified by gel extraction do not trigger cellular RNA sensors and their downstream innate immune-signaling pathways when transfected in cell culture.

## Discussion

As a potential therapeutic tool, circRNAs have attracted the attention of many scientists in recent years. This has sparked a discussion about the immunogenicity of circRNAs.^33^^,^^34^ Initially reported in 2017, Chen et al.^33^ proposed that exogenous circRNAs, produced using a cell-free permuted intron-exon (PIE) system, led to potent immune activation when transfected into HeLa cells. Furthermore, they showed that the virus RNA sensor RIG-I is necessary and sufficient for sensing of exogenous circRNA and, contrary to previous data, rather independent of RNA 5′ triphosphate ends or blunt-ended double-stranded RNA.^41^ RIG-I recognition was thereby dependent on the intron that facilitates circularization, since RNA-binding proteins are deposited on the circRNA, reflecting its origin and biogenesis.^33^ In contrast, 2 years later, Wesselhoeft et al.^34^ demonstrated that exogenous, PIE-derived circRNAs are able to bypass RNA sensors, including RIG-I, thereby circumventing the induction of cellular antiviral defense mechanisms. They conclude that the initially described immunogenicity of circRNAs result from 5′-triphosphorylated linear RNA contaminants that induce innate immune responses through RIG-I and TLRs. In general, artificial circRNA-mediated immune activation critically relies on (1) the production strategy (*in vivo* or *in vitro*) and purification method or purity of the final product, (2) RNA modifications, (3) secondary structure, and (4) the dose of the circRNA administered. However, in-depth analyses of cellular fate and immunogenicity of relatively short, highly purified artificial circRNAs produced in cell-free systems are still lacking.

### Production strategies and purity of artificial circRNAs

To not only identify but also rather increase the functional understanding of circRNAs, a broad range of artificial circRNA expression systems was established. Relying on specifically designed overexpression vectors, circRNAs can be produced in cell culture (here termed “*in vivo*”) both by spliceosome-dependent exon circularization strategy^2^^,^^12^^,^^42^ and independent of the spliceosome based on engineered ribozymes derived from the tRNA splicing machinery (e.g., the “tornado” system).^43^ In addition, strategies relying on circRNA production via cell-free systems (here termed “*in vitro*”) using recombinant T7-phage RNA-polymerase-mediated transcription and circularization by either employing genetically engineered autocatalytic group I introns (e.g., the PIE system)^42^^,^^44^, ^45^, ^46^ or enzymatic intramolecular ligation using recombinant RNA ligases^8^^,^^9^^,^^20^^,^^42^ gained importance for further characterization of circRNA utilities. The increasing knowledge on production strategies of artificial circRNAs leads to a rapid development of the potential range of application—e.g., in the context of viral infections,^8^^,^^47^ cancer,^9^^,^^29^ and cardiovascular disease.^48^

In this respect, the purity of the isolated artificial circRNAs is crucial for avoidance of triggering innate immune responses with contaminants from the circRNA production process. In this study, we analyzed the cellular innate immune response of A549 cells treated with *in vitro* produced and polyacrylamide-urea-gel-purified artificial circRNA sponges. We have used the same circRNAs that were applied in Müller et al.,^9^ with miRNA-21-binding sites (“bulged” binding configuration, abbreviated “bu”) and a randomized control sequence based on the nucleotide composition of the miRNA-21 sponge construct (“rnd”). Interestingly, ciRS-21-rnd and ciRS-21-bu did not trigger the cellular innate immunity, as evident from mRNA levels of its downstream components. Although several GO terms related to immune activation are enriched, the selected candidate mRNAs show very moderate increase (2- to 4-fold) in differential expression (Figure 3) and in qRT-PCR analyses (Figures 5 and 6) when the circular RNAs ciRS-21-bu und ciRS-21-rnd are transfected, similar to mock (liposome-) transfected cells. In contrast, when the innate immune response is triggered by poly(I:C)-positive controls or a long double-stranded circRNA (ciRS-21-ds), candidate mRNA levels increase by 10- to 500-fold in a time- and dose-dependent manner (Figures 5 and 6). Therefore, we conclude that both ciRS-21-bu and ciRS-21-rnd are not immunogenic when produced by *in vitro* transcription and circularization and purified by gel purification, as described in detail by Breuer and Rossbach.^20^

While HPLC and preparative phosphatase RNase R digestion^49^ may increase the purity of the circRNA, gel purification appears the most efficient method to obtain homogeneity of the preparation. As shown in Figure 1B, the choice of specific polyacrylamide concentrations during the purification gel run can “shift” the circular product to a region that is spatially separated from any other molecule species. This excludes contamination by linear mono- and dimers that may contain 5′-triphosphate ends, as well as by aberrant transcription products caused by formation of dsRNA region^50^ as well as remaining protein or nucleoside triphosphates (NTPs) from the transcription reaction.

### RNA modifications

Furthermore, nucleoside isomers and RNA modifications in foreign RNAs are discussed in the context of immune-stimulatory or -suppressing effects. For instance, linear RNAs with nucleobase modifications like, e.g., pseudouridine or N1-methyl-pseudouridine did not stimulate TLR3, TLR7, and/or TLR8, in contrast to non-modified RNAs.^51^^,^^52^ Such approach of masking an RNA therapeutic from the innate immune system has successfully been used in the severe acute respiratory syndrome coronavirus 2 (SARS-CoV-2) mRNA vaccines (summarized in Morais et al.^32^).

In 2019, Chen et al.^53^ identified *N*^6^-methyladenosine (m^6^A) as marker of cellular circRNAs, which do not induce the known innate immune pathways, whereas unmodified circRNAs activated RIG-I and innate immune signaling. Interestingly, in a cell-free system for ciRS production by *in vitro* transcription, ligation, and stringent purification via gel extraction, and the given length of about 200 nt, our ciRSs (ciRS-21-bu and ciRS-21-rnd) were found to be non-immunogenic, even without any RNA modifications. This may be different with longer RNAs that are, e.g., constructed for translation purposes and not only miRNA sequestration. Nonetheless, when necessary for longer circRNAs, modified NTPs (e.g., pseudo-UTP) can be used in *in vitro* transcription reactions, replacing the respective unmodified NTP, as it is common practice in mRNA vaccine production.

### Secondary structure

Surprisingly, the highly double-stranded construct ciRS-21-ds (with a 50-bp perfectly double-stranded region) did not only upregulate the analyzed chemokine-, interferon-, and other innate-immune-response-associated mRNAs but also PKR, a key RNA sensor in cellular immunity. The autophosphorylation of threonine at position 446 in PKR is an indicator of PKR dimerization and activation.^54^ The latter suggests that sequence composition and especially extensive secondary structure elements may play a role in induction of the antiviral defense, since the ciRS-21-ds harboring the long double-stranded region not only fails to bypass detection by cellular innate immunity but even induces the latter to a comparable extent as the poly(I:C) dsRNA controls.

In summary, the entirety of all properties of an artificial circRNA may be crucial regarding the recognition and RNA-sensor-mediated immune activation and thus the cellular fate of a potential therapeutic—from design, production strategy, and stringent purification to the extent of double-stranded regions in a ciRS. The latter provides opportunities and possibilities for immunomodulatory effects. Thus, it could be useful in certain applications to either specifically trigger or bypass the innate immune system with an artificial circRNA therapeutic.

## Materials and methods

### ciRS design and production

The circRNAs used here were designed as initially described in Jost et al.^8^ and contain a region at both ends enabling formation of an 11-nt stem region with a 10-nt open loop. Located in proximity of the stem loop, a 63-nt constant region is shared between all constructs and serves as a binding site for northern-detection probes and PCR primers. The miRNA sponge construct ciRS-21-bu carries four consecutive miR-21-binding sites, each separated by a 4-nt spacing. Note that the terminal 5′- and 3′ nt of miRNA-21 cannot base pair with the sponge sequence. Also, nucleotides 11–13, directly adjacent to the “seed” sequence of the miRNA, cannot base pair and form the eponymous “bulge.” The construct ciRS-21-rnd is characterized by the same nucleotide composition as ciRS-21-bu, but instead of miRNA-21-binding sites, it harbors a randomized negative control sequence. Identical circRNA sponges were used in Müller et al.^9^ and are described in detail in Breuer et al.^20^ Based on these ciRSs, within this study, we also designed a highly double-stranded circRNA sponge containing a randomized self-complementary sequence of 50 bp: ciRS-21-ds.

Template sequences were chemically synthesized and cloned into the multipurpose vector backbone containing the sequence elements described above, excised by flanking *Eco*RI restriction sites and gel purified via agarose gel electrophoresis. The purified template was used for *in vitro* transcription using the HiScribe T7 High Yield RNA Synthesis Kit (New England Biolabs; cat. no. E2040S) with additional 10-fold molar excess of guanosine monophosphate (GMP) over NTPs (Merck KGaA; cat. no. G8377). Transcripts were treated by RQ1 DNase (Promega; cat. no. M6101) digestion to remove the DNA template. Next, excess NTPs were removed by size-exclusion chromatography (mini Quick Spin RNA Columns; Merck KGaA; cat. no. 11814427001). *In vitro* circularization was performed at 16°C overnight using the T4 RNA Ligase I (Thermo Fisher Scientific; cat. no. EL0021). Ligation reaction was analyzed on analytic 6%, 7%, and 8% polyacrylamide-urea gels enabling distinguishment of linear and circular products. Using preparative 7% polyacrylamide-urea gels, circular and linear sponges (ciRS/liRS) were visualized and excised using UV shadowing and purified by gel extraction within PK Buffer at 50°C. Phenol and chloroform extraction was performed to purify RNA followed by ethanol precipitation. Purified sponges were analyzed on 6%, 7%, and 8% polyacrylamide-urea gels as described above. Find detailed information and protocol elsewhere.^20^

### RNase R treatment

Circularity was proven by RNase R treatment. Therefore, 200 ng of the RNA was incubated with 2 U RNase R (Lucigen; cat. no. RNR07250) for 30 min at 37°C. Afterward, 50% of the reaction was analyzed via polyacrylamide-urea gel electrophoresis and ethidium bromide staining.

### Cell culture and transfection

A549 cells were cultured in Dulbecco’s modified Eagle medium supplemented with 10% fetal bovine serum at 37°C and 5% CO_2_. Twenty-four hours prior transfection 4.2 × 10^5^ cells were seeded on 6-well plates. The transfection of cells was performed using Lipofectamine 2000 (Thermo Fisher Scientific; cat. no. 11668019) according to manufacturer’s instructions. Depending on the approach, 21 ng to 7 μg of the circular RNA sponges, LMW poly(I:C) (InvivoGen; cat. no. tlrl-picw), HMW poly(I:C) (InvivoGen; cat. no. tlrl-pic), or single-stranded (ss) PolyU (InvivoGen; cat. no. tlrl-lpu) were added to the transfection reaction. If not harvested beforehand, cells were washed with PBS and medium was exchanged 4 h post-transfection.

### RNA isolation and qRT-PCR

Cells were lysed and total RNA was isolated using TRIzol (Thermo Fisher Scientific; cat. no. 15596026) reagent. Two micrograms of total RNA served as template for reverse transcription using the qScript cDNA Synthesis Kit (QuantaBio; cat. no. 733-1174) according to manufacturer’s protocols. Two-step qRT-PCR with an annealing temperature of 58°C was performed utilizing Luna Universal qPCR Master Mix (New England Biolabs; cat. no. M3003E) based on SYBR green technology in both a QuantStudio 3 and StepOnePlus Real-Time PCR cycler (Thermo Fisher Scientific). Primer pairs were selected using the Primer Blast designing tool (https://www.ncbi.nlm.nih.gov/tools/primer-blast/). Relative quantification of mRNA abundance was ascertained as described by Michael W. Pfaffl^55^ using the mean of housekeeping genes U1 small nuclear RNA (snRNA), U6 snRNA, and B2M for normalization. Primers used are listed in Table S1.

### Sequencing and bioinformatic analysis

RNA was isolated as described above, and library preparation was performed using SMARTer Stranded Total RNA Sample Prep Kit (Takara; cat. no. 634873), which includes rRNA removal. Sequencing was performed using a NextSeq 500 High Output Kit (75 cycles).

#### Data preprocessing

The quality of the datasets was examined with fastqc v.0.11.9 (https://www.bioinformatics.babraham.ac.uk/projects/fastqc/; accessed on 22 June 2020) before and after trimming. Adapters were trimmed using Trim_Galore v.0.6.4 (https://www.bioinformatics.babraham.ac.uk/projects/trim_galore/; accessed on 22 June 2020), which used cutadapt v.2.8,^56^ removing all reads shorter than 30 nt after the adapter removal (settings: --length 30). Nucleotides with a phred score below 20 were removed using fastq_quality_filter from the FASTX toolkit v.0.0.14 (https://github.com/agordon/fastx_toolkit; accessed on 22 June 2020), also removing reads that became shorter than 90% of their original length (settings: -q 20 -p 90). The remaining reads were aligned to the human genome (GRCh38) with the associated annotation from Ensembl^57^ using STAR v.2.7.3a^58^ with standard settings. All subsequent analyses were executed in R version 3.6.3.^59^

#### Differential gene expression analysis

Alignments were assigned to their feature via featureCounts from the Rsubread package v.2.2.6.^60^ The resulting count matrix was used for the differential gene expression analysis via the DESeq2 package v.1.28.1.^61^ Genes with a p < 0.1 and a log2FoldChange lower than −2 or higher than 2 (4-fold increase or decrease) were considered to be differentially expressed.

#### GO term analysis

IDs of differentially expressed genes were converted from ensembl to entrez format via the package org.Hs.eg.db v.3.11.4,^62^ and GO terms were assigned to each gene using goana from the R package limma version 3.44.3.^63^ For a better overview, the amount of GO terms was reduced by using owltools version 2020-04-06 (https://github.com/owlcollab/owltools; accessed on 25 June 2020) with the generic GO subset from the Gene Ontology Resource.^64^ For visualization, the R package ggplot2 version 3.3.5^65^ was used. Total RNA sequencing data were deposited at NCBI GEO (GEO: GSE192656).

### Western blot

Cells were harvested by scraping, and total protein was extracted using PBS containing 0.5% Triton X-100. Proteins were separated via SDS-PAGE, containing 4% polyacrylamide stacking gel and 12% polyacrylamide resolving gel, and transferred to PVDF Western Blotting Membrane (Roche Consumer Health Deutschland; cat. no. 030100400001). Specific primary antibodies binding to p-PKR (Thr446) (Abcam; cat. no. ab32036), PKR (Proteintech; cat. no. 18244-1-AP), and γ-tubulin (Sigma-Aldrich; cat. no. T6557) followed by horseradish-peroxidase-coupled secondary antibodies (anti-mouse-immunoglobulin G [IgG], Sigma-Aldrich, cat. no. A9917; anti-rabbit-IgG: Sigma-Aldrich, cat. no. A0545) were used to determine the protein abundance. Visualization was achieved utilizing luminol-based chemiluminescent substrates Lumi-Light Western Blotting Substrate (Roche Consumer Health Deutschland; cat. no. 12015200001) and Lumi-Light Plus Western Blotting Substrate (Roche Consumer Health Deutschland; cat. no. 12015196001) as well as INTAS ECL Chemocam Imager (Intas Science Imaging Instruments).

## Supplemental information

Document S1. Tables S1–S6

Document S2. Article plus supplemental information
