# Supplementary material for: What goes around comes around: Artificial circular RNAs bypass cellular antiviral responses
Source: Mol Ther Nucleic Acids. 2022 Apr 27;28:623–35. doi: 10.1016/j.omtn.2022.04.017 (PMC9042720; doi:10.1016/j.omtn.2022.04.017)
Supplement: Document S1. Tables S1–S6 [file mmc1.pdf]

OMTN, Volume *28*

Supplemental information

What goes around comes around: Artificial circular RNAs bypass cellular antiviral responses

Janina Breuer, Patrick Barth, Yannic Noe, Lyudmila Shalamova, Alexander Goesmann, Friedemann Weber, and Oliver Rossbach

SUPPLEMENTAL INFORMATION

Table S1: RT-qPCR Primer used.

Primer	Sequence (5´-3´)
CXCL2_fwd<br>CXCL2_rev	CACAGTGGCTGACATGTGATA<br>AGGTCAAACCCAAGTTAGTTCA
CXCL3_fwd<br>CXCL3_rev	GAGCCGGGGATTGCTGG<br>CTCTCCCGCTTCTCGCAC
CXCL8_fwd<br>CXCL8_rev	GGTGCAGTTTTGCCAAGGAG<br>TGGGGTGGAAAGGTTTGGAG
CXCL10_fwd<br>CXCL10_rev	AGGAACCTCCAGTCTCAGCA<br>CAAAATTGGCTTGCAGGAAT
EGR1_fwd<br>EGR1_rev	CAGGTCAGCAGCTTCCCTTC<br>TCATGTCCGAAAGCCCTGTG
INFB1_fwd<br>INFB1_rev	TGGGAGGATTCTGCATTACC<br>CAGCATCTGCTGGTTGAAGA
IRAK2_fwd<br>IRAK2_rev	GGACCCTTGTCCTCAGTTGG<br>CACCCAGGACAGCAGATGTT
MDA-5_fwd<br>MDA-5_rev	GCATATGCGCTTTCCCAGTG<br>CTCTCATCAGCTCTGGCTCG
OAS1_fwd<br>OAS1_rev	TGGATTCTGCTGGTGAGACC<br>ATGGCCTTTGGCAAGAGGTAAG
PKR_fwd<br>PKR_rev	TCCATGGGGAATTACATAGGC<br>AGCGGCCAATTGTTTTGCTT
RIG-I_fwd<br>RIG-I_rev	AAATCAGAACACAGGCAGAGGAA<br>GTCCCATGTCTGAAGGCGTAA

TLR3_fwd<br><br>TLR3_rev	CCTTTTGCCCTTTGGGATGC<br><br>TGAAGTTGGCGGCTGGTAAT
TLR7_fwd<br><br>TLR7_rev	CCTTGTGCGCCGTGTAAAAA<br><br>GGGCACATGCTGAAGAGAGT
TLR8_fwd<br><br>TLR8_rev	CCAAACTGCCAAGCTCCCTA<br><br>CAGCACCTTCAGATGAGGCA
TRAF1_fwd<br><br>TRAF1_rev	GGTGCAGGTGTCAATGAAGC<br><br>ACAAGCCCCCATGAGAAACA

Table S2: P-values of CXCL2, CXCL3, CXCL8, EGR1 and TRAF1 in context of the time dependent immunogenicity to ciRS-21-ds and poly(I:C) in contrast to ciRS-21-rnd and ciRS-21-bu treatment. Statistical significance was determined by Student's t-test. White background p-value < 0.05, significant; grey background p-value > 0.05, not significant.

		CXCL2	CXCL3	CXCL8	IRAK2	EGR1	TRAF1
0 h	mock						
	ciRS-21-bu	0.09	0.06	0.84	8.42E-03	0.72	0.86
	ciRS-21-rnd	3.09E-02	5.01E-02	0.59	0.16	0.32	0.46
	ciRS-21-ds	0.58	0.44	0.64	0.25	0.53	0.11
	LMW Poly(I:C)	2.46E-02	0.14	0.17	5.93E-03	0.84	0.65
	HMW Poly(I:C)	5.43E-02	0.33	0.24	0.13	0.91	0.35
0.5 h	mock						
	ciRS-21-bu	0.15	4.50E-02	0.09	0.83	1.94E-09	0.28
	ciRS-21-rnd	2.89E-02	6.62E-03	4.69E-03	0.37	1.06E-09	0.20
	ciRS-21-ds	8.23E-04	2.05E-04	3.18E-04	8.10E-03	8.82E-12	0.23
	LMW Poly(I:C)	8.11E-03	4.08E-03	1.83E-03	0.06	2.74E-11	0.19
	HMW Poly(I:C)	0.18	0.06	0.11	0.28	2.56E-10	0.49
2 h	mock						
	ciRS-21-bu	0.75	0.11	0.18	1.90E-02	0.43	0.55
	ciRS-21-rnd	0.70	0.27	4.85E-03	0.55	3.38E-04	1.45E-02
	ciRS-21-ds	5.32E-06	0.82	1.12E-05	1.25E-03	1.07E-09	2.80E-05
	LMW Poly(I:C)	3.91E-07	2.05E-04	6.45E-10	6.25E-04	1.84E-12	1.09E-06
	HMW Poly(I:C)	2.12E-07	1.13E-02	2.46E-08	2.93E-03	1.34E-13	2.94E-07
3 h	mock						
	ciRS-21-bu	0.10	0.53	0.82	8.09E-03	6.88E-03	0.79
	ciRS-21-rnd	0.28	1.24E-03	6.91E-01	0.61	3.94E-02	0.53
	ciRS-21-ds	8.46E-08	5.24E-06	1.32E-07	0.22	1.25E-12	2.44E-06
	LMW Poly(I:C)	1.17E-07	2.65E-06	1.05E-09	0.61	2.22E-14	2.45E-09
	HMW Poly(I:C)	7.29E-09	1.66E-06	4.48E-09	0.17	2.81E-13	1.50E-09
4 h	mock						
	ciRS-21-bu	0.15	1.22E-02	0.48	0.96	2.26E-02	0.54
	ciRS-21-rnd	3.63E-05	2.05E-03	1.06E-03	1.91E-02	0.06	2.43E-03
	ciRS-21-ds	3.39E-09	3.55E-05	5.29E-07	4.55E-02	1.02E-13	7.00E-09
	LMW Poly(I:C)	3.84E-10	4.37E-06	1.22E-08	0.16	1.59E-13	3.52E-09
	HMW Poly(I:C)	2.35E-11	1.30E-08	7.40E-07	0.06	2.01E-13	1.22E-09
5 h	mock						
	ciRS-21-bu	0.09	3.31E-03	0.78	2.41E-02	4.26E-02	0.39
	ciRS-21-rnd	0.08	0.06	0.75	0.25	1.43E-02	0.31
	ciRS-21-ds	1.74E-09	1.30E-07	3.96E-06	4.88E-05	1.70E-14	1.40E-08
	LMW Poly(I:C)	2.99E-12	4.02E-10	6.54E-07	5.53E-04	1.39E-14	1.43E-08
	HMW Poly(I:C)	2.60E-10	1.52E-08	7.37E-07	6.61E-06	3.70E-15	6.03E-09
12 h	mock						
	ciRS-21-bu	5.71E-04	8.62E-05	0.97	0.16	0.16	0.95
	ciRS-21-rnd	0.53	3.96E-03	0.69	0.58	0.85	0.80
	ciRS-21-ds	7.55E-08	1.58E-10	1.25E-04	3.59E-02	2.50E-12	2.66E-08
	LMW Poly(I:C)	4.74E-07	1.48E-11	9.28E-05	1.07E-03	5.64E-13	6.31E-09
	HMW Poly(I:C)	7.44E-08	1.27E-11	5.39E-06	2.11E-03	3.84E-13	3.93E-09
24 h	mock						
	ciRS-21-bu	9.79E-05	0.93	0.34	0.97	4.62E-02	0.15
	ciRS-21-rnd	6.21E-04	3.47E-04	0.87	0.93	2.83E-03	0.36
	ciRS-21-ds	9.45E-05	8.07E-10	5.34E-05	0.51	9.27E-09	1.42E-02
	LMW Poly(I:C)	8.00E-06	1.98E-10	1.76E-06	0.12	1.59E-09	4.23E-08
	HMW Poly(I:C)	9.65E-05	5.30E-09	4.47E-06	0.17	8.18E-10	8.85E-07

Table S3: P-values of CXCL10 and INFB1 in context of the time dependent immunogenicity to ciRS-21-ds and poly(I:C) in contrast to ciRS-21-rnd and ciRS-21-bu treatment. Statistical significance was determined by Student's t-test. White background p-value < 0.05, significant; grey background p-value > 0.05, not significant.

		CXCL10	INFB1
0 h	mock		
	ciRS-21-bu	0.87	0.79
	ciRS-21-rnd	0.86	0.72
	ciRS-21-ds	0.92	0.69
	LMW Poly(I:C)	0.89	0.94
	HMW Poly(I:C)	0.86	0.59
0.5 h	mock		
	ciRS-21-bu	0.77	0.93
	ciRS-21-rnd	0.96	0.94
	ciRS-21-ds	0.82	0.55
	LMW Poly(I:C)	0.76	0.81
	HMW Poly(I:C)	0.82	0.33
2 h	mock		
	ciRS-21-bu	0.97	7.58E-04
	ciRS-21-rnd	0.86	0.89
	ciRS-21-ds	0.90	3.45E-05
	LMW Poly(I:C)	3.16E-05	5.63E-10
	HMW Poly(I:C)	3.31E-05	5.84E-10
3 h	mock		
	ciRS-21-bu	0.89	0.40
	ciRS-21-rnd	0.61	0.63
	ciRS-21-ds	0.83	3.09E-03
	LMW Poly(I:C)	1.79E-06	9.96E-14
	HMW Poly(I:C)	4.14E-06	1.05E-13
4 h	mock		
	ciRS-21-bu	0.90	0.37
	ciRS-21-rnd	0.97	0.74
	ciRS-21-ds	2.14E-02	6.62E-05
	LMW Poly(I:C)	1.17E-10	1.85E-12
	HMW Poly(I:C)	6.39E-11	3.73E-04
5 h	mock		
	ciRS-21-bu	0.79	0.81
	ciRS-21-rnd	0.96	0.95
	ciRS-21-ds	0.24	4.69E-04
	LMW Poly(I:C)	1.14E-08	1.65E-13
	HMW Poly(I:C)	2.70E-08	2.16E-05
12 h	mock		
	ciRS-21-bu	0.72	2.69E-05
	ciRS-21-rnd	1.00	0.97
	ciRS-21-ds	1.67E-02	1.98E-06
	LMW Poly(I:C)	1.18E-07	8.16E-16
	HMW Poly(I:C)	2.99E-07	9.05E-05
24 h	mock		
	ciRS-21-bu	1.00	0.19
	ciRS-21-rnd	0.99	0.61
	ciRS-21-ds	8.41E-04	2.26E-03
	LMW Poly(I:C)	1.55E-08	6.10E-08
	HMW Poly(I:C)	8.72E-08	1.32E-06

Table S4: P-values of CXCL2, CXCL3, CXCL8, EGR1 and TRAF1 in context of the dose dependent immunogenicity to ciRS-21-ds and poly(I:C) in contrast to ciRS-21-rnd and ciRS-21-bu treatment. Statistical significance was determined by Student's t-test. White background p-value < 0.05, significant; grey background p-value > 0.05, not significant.

		CXCL2	CXCL3	CXCL8	EGR1	IRAK2	TRAF1
	untreated mock	3.13E-03	1.37E-02	0.76	4.30E-05	1.51E-02	1.48E-03
21 ng	ciRS-21-bu	2.74E-03	0.79	0.11	5.36E-04	2.84E-02	3.04E-04
	ciRS-21-rnd	1.38E-03	0.23	0.13	0.59	0.06	4.25E-04
	ciRS-21-ds	6.38E-06	4.32E-07	1.36E-03	2.63E-08	2.44E-02	8.66E-07
	LMW Poly(I:C)	8.24E-07	8.62E-08	1.99E-05	4.53E-10	1.62E-03	2.17E-08
	HMW Poly(I:C)	3.49E-07	4.19E-08	3.61E-05	4.75E-11	2.12E-03	5.07E-10
105 ng	ciRS-21-bu	6.20E-03	0.98	2.26E-02	0.38	6.57E-03	2.39E-04
	ciRS-21-rnd	8.89E-04	0.10	1.39E-02	0.83	1.72E-02	6.62E-07
	ciRS-21-ds	7.02E-07	3.85E-07	1.74E-04	5.10E-10	2.03E-03	8.89E-08
	LMW Poly(I:C)	2.11E-07	3.60E-09	7.68E-05	1.82E-11	9.33E-03	9.59E-11
	HMW Poly(I:C)	1.36E-07	5.62E-08	8.22E-06	1.71E-11	0.24	1.25E-09
250 ng	ciRS-21-bu	1.08E-03	7.41E-03	4.25E-02	8.69E-04	0.09	8.87E-06
	ciRS-21-rnd	2.86E-03	0.11	0.79	1.05E-02	0.26	4.40E-05
	ciRS-21-ds	1.52E-07	2.89E-08	1.30E-05	3.16E-11	1.04E-02	1.30E-09
	LMW Poly(I:C)	1.15E-07	2.16E-09	8.77E-05	5.65E-12	3.34E-02	5.22E-11
	HMW Poly(I:C)	7.21E-08	1.76E-08	1.96E-06	2.01E-11	9.64E-02	2.22E-09
700 ng	ciRS-21-bu	6.08E-05	9.63E-06	0.14	1.20E-03	7.89E-03	4.49E-06
	ciRS-21-rnd	8.62E-04	2.43E-02	0.07	0.59	0.09	2.04E-03
	ciRS-21-ds	3.95E-09	4.41E-10	9.71E-08	5.27E-13	2.44E-04	4.16E-11
	LMW Poly(I:C)	1.32E-07	4.88E-09	1.70E-05	2.02E-12	0.39	8.12E-10
	HMW Poly(I:C)	6.02E-07	4.97E-08	4.46E-06	2.39E-12	0.08	6.27E-10
3500 ng	ciRS-21-bu	2.06E-05	1.37E-05	3.99E-02	7.91E-05	7.21E-03	1.79E-05
	ciRS-21-rnd	6.43E-04	0.30	0.74	0.13	9.50E-03	4.43E-05
	ciRS-21-ds	3.11E-09	2.69E-10	4.38E-08	4.73E-13	2.04E-03	8.44E-11
	LMW Poly(I:C)	1.87E-07	4.76E-09	1.18E-05	1.41E-12	0.34	1.82E-09
	HMW Poly(I:C)	6.23E-07	4.08E-08	6.13E-05	3.39E-12	2.36E-02	1.03E-09
7000 ng	ciRS-21-bu	2.52E-05	9.04E-06	1.10E-03	6.85E-05	6.71E-03	6.45E-07
	ciRS-21-rnd	9.57E-04	0.10	0.79	0.26	1.91E-03	3.15E-04
	ciRS-21-ds	9.46E-10	4.42E-08	8.48E-07	9.60E-13	1.40E-04	5.80E-11
	LMW Poly(I:C)	1.86E-07	1.87E-08	7.26E-05	1.10E-12	4.27E-02	6.43E-09
	HMW Poly(I:C)	1.15E-07	1.50E-08	1.77E-05	7.03E-12	0.11	1.05E-08

Table S5: P-values of CXCL10 and INFB1 in context of the dose dependent immunogenicity to ciRS-21-ds and poly(I:C) in contrast to ciRS-21-rnd and ciRS-21-bu treatment. Statistical significance was determined by Student's t-test. White background p-value < 0.05, significant; grey background p-value > 0.05, not significant.

		CXCL10	INFB1
	untreated		
	mock	0.18	0.20
21 ng	ciRS-21-bu	0.30	7.97E-03
	ciRS-21-rnd	7.07E-03	0.24
	ciRS-21-ds	3.68E-04	0.09
	LMW Poly(I:C)	7.78E-11	1.39E-30
	HMW Poly(I:C)	1.20E-10	8.67E-32
105 ng	ciRS-21-bu	0.56	5.16E-02
	ciRS-21-rnd	0.48	6.99E-04
	ciRS-21-ds	3.56E-04	3.03E-05
	LMW Poly(I:C)	2.73E-13	2.18E-43
	HMW Poly(I:C)	3.65E-13	2.09E-45
250 ng	ciRS-21-bu	0.90	4.69E-03
	ciRS-21-rnd	0.27	0.12
	ciRS-21-ds	5.39E-06	4.24E-08
	LMW Poly(I:C)	1.57E-14	9.10E-51
	HMW Poly(I:C)	1.52E-14	7.73E-54
700 ng	ciRS-21-bu	1.85E-05	2.70E-04
	ciRS-21-rnd	8.31E-04	0.45
	ciRS-21-ds	4.43E-09	1.98E-20
	LMW Poly(I:C)	3.78E-15	8.24E-58
	HMW Poly(I:C)	1.07E-15	4.36E-63
3500 ng	ciRS-21-bu	1.12E-06	2.40E-10
	ciRS-21-rnd	1.55E-04	8.45E-03
	ciRS-21-ds	3.14E-11	3.88E-33
	LMW Poly(I:C)	1.33E-15	4.40E-62
	HMW Poly(I:C)	1.75E-15	2.86E-65
7000 ng	ciRS-21-bu	3.68E-06	6.80E-12
	ciRS-21-rnd	1.22E-04	3.61E-05
	ciRS-21-ds	3.10E-11	8.45E-32
	LMW Poly(I:C)	1.42E-15	3.67E-62
	HMW Poly(I:C)	2.02E-14	6.12E-57

Table S6: P-values of candidate mRNAs 3h after A549 treatment with 250 ng of ciRS-21-ds and poly(I:C) in contrast to ciRS-21-rnd and ciRS-21-bu. Statistical significance was determined by Student's t-test for (**A**) CXCL2, CXC3, CXCL8, EGR1, IRAK2 and TRAF1, (**B**) CXCL10 and INFB1 as well as (**C**) MDA5, OAS1, PKR, RIG-I, TLR3, TLR7 and TLR8. White background p-value < 0.05, significant; grey background p-value > 0.05, not significant.

A

	CXCL2	CXCL3	CXCL8	EGR1	IRAK2	TRAF1
untreated						
mock	0.071	0.022	0.001	0.916	0.255	0.158
ciRS-21-bu	0.165	0.025	0.006	0.058	0.221	0.540
ciRS-21-rnd	0.127	0.264	0.027	0.089	0.946	0.477
ciRS-21-ds	1.76E-07	1.78E-08	9.58E-08	3.20E-10	0.63	2.90E-06
LMW Poly(I:C)	1.61E-08	1.70E-09	7.28E-09	2.98E-12	0.27	8.68E-08
HMW Poly(I:C)	2.08E-08	2.72E-10	1.30E-09	1.67E-12	0.16	8.40E-09
ssPolyU	0.211	0.701	0.025	0.028	0.042	0.007

B

	CXCL10	INFB1
untreated		
mock	0.188	0.224
ciRS-21-bu	0.291	0.092
ciRS-21-rnd	0.833	0.942
ciRS-21-ds	5.81E-05	2.62E-03
LMW Poly(I:C)	3.33E-14	1.15E-12
HMW Poly(I:C)	2.40E-14	1.35E-12
ssPolyU	0.096	0.031

C

	MDA5	OSA1	PKR	RIG-I	TLR3	TLR7	TLR8
untreated							
mock	0.07	0.86	0.64	0.60	8.54E-06	0.22	0.94
ciRS-21-bu	0.92	0.84	0.57	0.40	0.17	0.74	0.93
ciRS-21-rnd	0.92	0.27	0.89	0.99	6.14E-06	1.89E-03	4.23E-03
ciRS-21-ds	4.55E-02	7.42E-04	2.25E-02	1.20E-02	2.57E-05	6.89E-03	1.58E-03
LMW Poly(I:C)	8.32E-12	2.83E-03	0.10	7.10E-07	0.18	5.44E-05	3.93E-04
HMW Poly(I:C)	8.55E-11	9.31E-04	0.11	2.33E-06	9.73E-04	2.39E-04	1.33E-03
ssPolyU	0.86	0.59	0.52	0.53	4.02E-05	1.77E-03	1.10E-03
